# Conceptual Model for Using Imidazoline Derivative Solutions in Pulpal Management

**DOI:** 10.3390/jcm10061212

**Published:** 2021-03-15

**Authors:** Robert S. Jones

**Affiliations:** Division of Pediatric Dentistry, Department of Developmental & Surgical Sciences, School of Dentistry, University of Minnesota, Minneapolis, MN 55455, USA; rsjones@umn.edu

**Keywords:** hemostasis, alpha-adrenergic agonists, imidazoline, oxymetazoline, nasal, dental pulp, mucosa, apexogenesis, pulpotomy, direct pulp cap, dentistry

## Abstract

Alpha-adrenergic agonists, such as the Imidazoline derivatives (ImDs) of oxymetazoline and xylometazoline, are highly effective hemostatic agents. ImDs have not been widely used in dentistry but their use in medicine, specifically in ophthalmology and otolaryngology, warrants consideration for pulpal hemostasis. This review presents dental healthcare professionals with an overview of ImDs in medicine. ImD solutions have the potential to be more effective and biocompatible than existing topical hemostatic compounds in pulpal management. Through a comprehensive analysis of the pharmacology of ImDs and the microphysiology of hemostasis regulation in oral tissues, a conceptual model of pulpal management by ImD solutions is presented.

## 1. Overview

The purpose of this review is to formulate a conceptual model on the potential management of pulpal tissue by imidazoline derivatives (ImDs) based on a review of the literature that examines the hemostatic properties and mechanistic actions of these compounds in other human tissues. Commercial ImDs are formulated in solution with antimicrobial preservatives in order to act as ‘parenteral topical agents’ and used to manage ophthalmic inflammation, nasal congestion, and to control bleeding during otolaryngology surgery [1,2]. While systematic reviews of IMDs in medicine are published [3,4,5] this critical review proposes an advancement in dental pulpal management by examining the pharmacology and biochemistry of ImDs, which have been largely understudied for direct dental application [6,7,8,9]. ImD solutions that also contain antimicrobial preservatives may be more effective and biocompatible than existing topical hemostatic compounds in dentistry. These medicinal parenteral topical compounds have potential in pulpal management procedures such as a direct pulp cap, managing an exposure in a permanent tooth with an immature root (e.g., apexogenesis), and a primary tooth pulpotomy.

## 2. Imidazoline Derivatives

**Figure 1:**
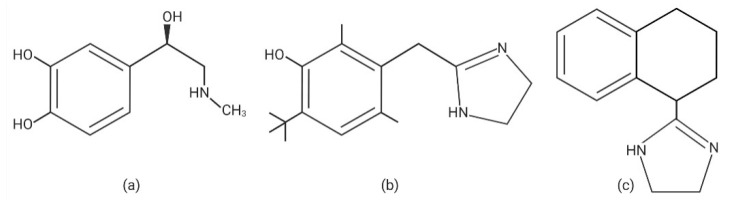
Catecholamines and Imidazoline Derivatives. (**a**) epinephrine with a root benzene ring (**b**) oxymetazoline has an imidazoline ring incorporated into the benzene ring structure (**c**) tetrahydrozoline (also known as tetryzoline) has an imidazoline ring bound to a polycyclic aromatic naphthalene.

There are several different medically useful ImDs compounds such as oxymetazoline, xylometazoline, naphazoline, and tetrahydrozoline (also known as tetryzoline). These ImDs are synthetic, noncatecholamines that can act as alpha adrenergic agonists. The root imidazoline ring makes them distinct from catecholamines such as epinephrine, norepinephrine, and phenylephrine (Figure 1). Oxymetazoline and xylometazoline are nearly identical in chemical structure with oxymetazoline having an additional hydroxyl group substitution. Naphazoline and tetrahydrozoline are nearly identical with the imidazoline bound to a polycyclic aromatic naphthalene. ImDs are commonly formulated in hydrochloride (HCL) salts which are light/heat stable, soluble in water, and nonreactive when mixed with other compounds such as local anesthetics, alcohol, and antimicrobial preservatives.

## 3. Pharmacology and Use

ImDs are sympathomimetic compounds that directly act on alpha-adrenergic receptors within the sympathetic nervous system [10]. ImDs mimic catecholamines, norepinephrine and epinephrine, but are more selective to alpha(α)-1 and alpha(α)-2 post synaptic receptors [11]. ImDs have very little to no β-adrenergic agonist or antagonist activity that stimulates systemic effects within heart and lung tissue [12]. ImDs, such as oxymetazoline and xylometazoline, have a high affinity for α-1a subtype receptors that are ubiquitous in the nasal mucosa and also bind to less common α-2 receptors within the tissue [11]. ImDs have similar effects within cornea vessels [13]. The immediate local effect is vascular smooth muscle contraction (vasoconstriction) in pre-capillary arterioles and post-capillary venules [14]. In the case of nasal congestion, vasoconstriction reduces tissue edema and improves nasal airway flow (decongestant) [4]. Topical ocular application of ImDs reduces eye redness (conjunctivitis) [15]. ImDs, such as oxymetazoline and xylometazoline, can also be used to improve tissue visualization by controlling hemostasis for reduction based surgery, grafting procedures, and prosthetic placement in otolaryngology [2,16] (Table 1).

## 4. Efficacy

Oxymetazoline and xylometazoline activity have considerably longer duration of activity (8 h versus 1 h) than other sympathomimetics, such as epinephrine and phenylephrine [17]. Oxymetazoline and xylometazoline are the most effective vasoconstrictor agents during ear, nose, and throat surgical procedures and can be delivered pre- and intra-operatively [18,19]. While this surgical use is an off-label application [2], oxymetazoline and xylometazoline are approved and widely available as over-the-counter medications for multi-dose nasal congestion due to the reduction in local upper airway edema via sympathomimetic vasoconstriction [4]. Naphazoline and tetrahydrozoline are found in approved ophthalmic topical solutions. They are rarely used for hemostasis in and during surgical procedures, but naphazoline and tetrahydrozoline are frequently used to treat conjunctivitis as a home therapy [13,20].

Efficacy differences between ImDs are connected to duration of action and potency. Cross examination between ImDs have largely centered around examining common weight-to-volume percentage (*w*/*v*%) formulations found in available commercial products. Initial efficacy, using studies of nasal patency measurements, is indistinguishable between common formulations of naphazoline (0.02%), tetrahydrozoline (0.1%), xylometazoline (0.1%), and oxymetazoline (0.05%) [21]. However, the hemostatic differences are less widely examined and there is considerable duration of activity differences between these two sets of ImDs. The structurally similar naphazoline and tetrahydrozoline have substantially shorter duration of activity than the similar xylometazoline and oxymetazoline [21]. Naphazoline and tetrahydrozoline do not continue to reduce swelling after 4 h of administration, whereas both xylometazoline and oxymetazoline have extended effects depending on the amount used and may be effective up to 8 h [21,22]. Formulations of 0.05% oxymetazoline and 0.1% xylometazoline have been shown to have similar clinical effectiveness in adults [23]. The lower concentration of oxymetazoline needed to produce a comparative result is indicative of its higher potency, which may be attributed to its substantially higher affinity for alpha-1a adrenoreceptors [11].

## 5. Systematic Safety and Contraindications

The use of ImDs is ubiquitous in otolaryngeal surgery, in both children and adults, especially when excessive bleeding compromises the visual field [2]. Well planned studies on the pharmacokinetics, such as distribution and elimination, and dosing of these compounds are lacking. Current evidence suggests that controlled dosage of ImDs, such as light soaking of nasal pledgets, is safe and preferred over other vasoconstrictors, such as catecholamines, for surgical hemostasis in children [2,24]. Blood pressure, heart rate, and respiratory rates are well maintained in children during otolaryngology surgeries when ImDs dosing is premeasured [1,25,26]. It has been postulated that defined volume soaked pledgets of oxymetazoline held against the nasal mucosa cause such profound vasoconstriction that the drug has very slow absorption and limited systemic effects [1,27].

There is not a standardized maximum dosage of ImDs in parenteral topical applications. ImDs are lipophilic and while they have primarily local sympathomimetic activity, overdose of these compounds can lead to systemic absorption and activity including passing the blood–brain barrier. Surgical complications with ImDs have been documented in children and adults and are often associated with unmeasured soaking of nasal pledgets or inverted nasal spraying likely leading to overdose on healthy children or in individuals with complex medical conditions [28,29,30].

Systemic side effects have been seen in cases associated with home-use overdose in both young children (5 years and younger) and medically complex adults [30,31,32,33,34]. The over-the-counter availability of these solutions can circumvent medical professional warnings to avoid ImDs for home use in medically complex individuals and young children (5 years and younger) since it is difficult to correctly dose these individuals with spray bottles that can differ in volume delivery due to patient positioning [30,31,35]. Specific medical contraindications have not been determined, but there are medical precautions and drug class interactions where ImD may increase blood pressure causing unwanted effects (Table 2). There is also a caution when used during pregnancy and lactation. The medical precautions and drug interactions relate mainly to adults with medical conditions that would require medical consultation for dental treatment and local anesthesia/epinephrine administration.

Precaution should not be interpreted as contraindications given the safety of controlled dosing. For example, a single center, randomized, double-blind, placebo controlled trial from the Mayo clinic determined that adult patients with hypertension can be treated for epistaxis using standardized controlled dosing of 0.25% phenylephrine, 0.05% oxymetazoline, or 1% lidocaine with epinephrine 1:100,000 with no significant changes in heart rate and blood pressure (systolic and blood pressure) compared to a saline control [36].

Topical application to corneal tissue, comprising of distal tissue blood vessels, do not have published reports of adverse cardiovascular symptoms from direct ocular topical application, rather morbidities are related to unintentional ingestion [37,38]. Animal studies demonstrate that substantial chronic (3 × daily/4 weeks) use of ImDs may cause distal tissue (rat tail) necrosis from the systemic effects [39]. There is currently no evidence that a single application of ImDs can produce distal tissue necrosis, which would be a potential concern for use in pulpal hemostasis. Evidence that direct application of higher dose epinephrine is safe for digit surgery and does not lead to necrosis supports the single use of the general class of vasoconstrictive agents on distal end tissues such as pulpal tissue [39].

## 6. Anesthetic Adjunct for Intranasal Dental Anesthetic

ImDs have recently been tested to be delivered intranasally to assist in anesthetizing maxillary anterior teeth without the use of a needle [40]. Intranasal application of 0.05% oxymetazoline is used as an adjunct to 3% tetracaine to slow the systemic absorption of the anesthetic and improve the overall efficacy [40]. Maxillary teeth anesthesia is achieved by effectively blocking innervation of the branch of the infraorbital nerve that descends from the infraorbital foramen in the walls of the sinus before reaching the anterior maxillary teeth. The standard epinephrine that is often added to local anesthetics is substituted for oxymetazoline since the highly vascular nasal mucosa is difficult to extensively vasoconstrict with epinephrine alone. A randomized placebo controlled study of 150 adult subjects demonstrated that the intranasal delivery of a tetracaine/oxymetazoline formulation (Kovanaze™) produces 88% (95%CI 80–93.6) of upper maxillary teeth anesthesia via blockage of the anterior and middle alveolar nerve [9]. The study reported no serious adverse events [9]. The results of this trial, and a previous preliminary study, are surprisingly one of the few pharmacokinetics studies of oxymetazoline within medicine [9,40]. Cardiac parameters such as systolic, diastolic, and heart rate had minimal and clinically insignificant changes at a median dose of 0.0025–0.0037 mg kg^−1^ of oxymetazoline in adults [9].

## 7. Tissue Biocompatibility and Antimicrobial Activity

The biocompatibility, toxicity, and effects of ImDs on pulpal tissue and effects on caries pathogens are unknown at this time. Given that ImDs are inexpensive, over-the-counter medications, and off-patent, it is doubtful that in the USA that a dental company would seek approval to investigate an off-patent approach for pulpal toxicity and activity.

Despite this future limitation, there are studies that support overall tissue biocompatibility and antimicrobial activity. ImDs are labeled for nasal and ophthalmic use but not directly for ear treatments. Ear treatment is an off-label use in the USA. There are animal models and laboratory microbiology investigation on the ototoxicity and antimicrobial activity of oxymetazoline and tetrahydrozoline solutions [41,42]. The results of these animal and microbiology investigations are relatable to how ImD solutions may effect pulpal tissue. Oxymetazoline at the commercially available concentration of 0.05% was non-toxic to sensitive auditory hair cells in Guinea pigs [41,42]. Furthermore, this investigation found that pure oxymetazoline (at 0.05%) alone was not antimicrobial [41]. However, 0.05% oxymetazoline nasal spray and tetrahydrozoline ophthalmic eye drops are formulated with antimicrobial preservatives (e.g., benzalkonium chloride, edetate disodium-EDTA, alcohol) and these solutions have antimicrobial activity toward Gram positive bacteria (e.g., *Streptococcus pneumonia* and *Staphylococcus aureus*) [41]. The effects of nasal spray solutions on Gram negative was more varied but clinical results support broad antimicrobial effects [41]. A prospective cross-sectional study found that commercial 0.05% oxymetazoline solutions were equivalent to ciprofloxacin drops in preventing infection otorrhea following surgery without any toxic hearing loss complications [43]. These aforementioned animal model studies, in addition to a retrospective examination of over 10,000 treated ears with 0.05% oxymetazoline solutions without complications or hearing loss, supports the biocompatibility and lack of ototoxicity with ImD solutions [44]. Recent in vitro studies examining ImDs on primary human gingival fibroblasts for the prospect of an experimental gingival retraction agent also suggests high potential biocompatibility with oral tissues [7].

## 8. Pulpal Microphysiology and Management

**Figure 2:**
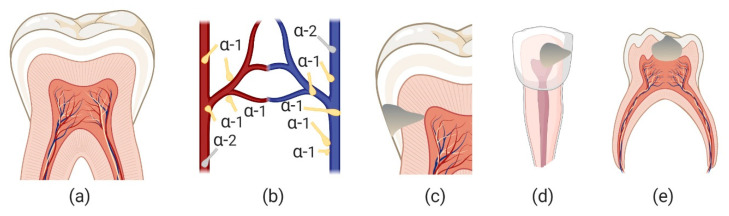
Tooth Anatomy and Pulpal Management. (**a**) A Cross-section of the pulp chamber with capillary plexus and post-capillary venules (**b**) sympathetic innervation is dominated by post synaptic α-1 adrenoreceptors associated with smooth muscles of arterioles and venules. A smaller population of the α-2 subtype is thought to exist (**c**) caries approaching pulp where mechanical exposure may require direct pulp capping (**d**) permanent tooth with immature roots and a caries exposure (or trauma exposure) that may require partial pulp removal for apexogenesis (**e**) large caries exposure on primary tooth requiring vital pulpotomy.

Pulpal blood flow originates mainly from arterioles entering the apical foramen. The arterioles branch out into a capillary plexus and then enters the post-capillary venules and exits the tooth through larger venules (Figure 2a).

The arterioles and venules are lined with smooth muscles that are mainly innervated by sympathetic nerves [43]. These unmyelinated nerve fibers possess α-1 adrenoreceptors with a much smaller number of α-2 adrenoreceptors (Figure 2b). Parasympathetic nerve innervation and vasodilation regulation of the vascular pulp tissue is absent or minor in animal pulpal studies [45,46]. Sympathetic mediated vasoconstriction in dental pulp cells is thought to be mediated by α-1 [45,47]. Exogenous topically applied vasoconstriction is needed in several situations of carious or traumatic exposure of pulpal tissue. In cases of smaller caries exposures in permanent teeth (Figure 2c), direct pulp capping procedures involve pulpal hemostasis, cavity conditioning, and biocompatible material placement (e.g., MTA) which can preserve pulpal vitality. It is important to consider that indirect pulp capping is most often the preferred procedure but small exposures can occur that require hemostasis [48]. The blood flow near the pulp horns is greater than any other region of the pulp [49], and pulpal hemostasis can be difficult to achieve. Hemostasis is also required for large carious or traumatic exposure in permanent teeth with immature root formation (Figure 2d). In these teeth, blood volume and flow is substantial given the open immature apical foremen [50]. In these cases, the objective is to achieve hemostasis and maintain radicular vitality (apexogenesis) by placing biocompatible material either partially or fully in the coronal pulp chamber. These are two examples of the need for effective permanent teeth pulpal hemostasis. For primary teeth, large caries exposures may require removal of the coronal pulp and hemostasis of the radicular pulp is necessary before a biocompatible material is placed in the chamber and followed by restorative placement (Figure 2e).

## 9. Conceptual Model of Pulpal Exposure Management

**Figure 3:**
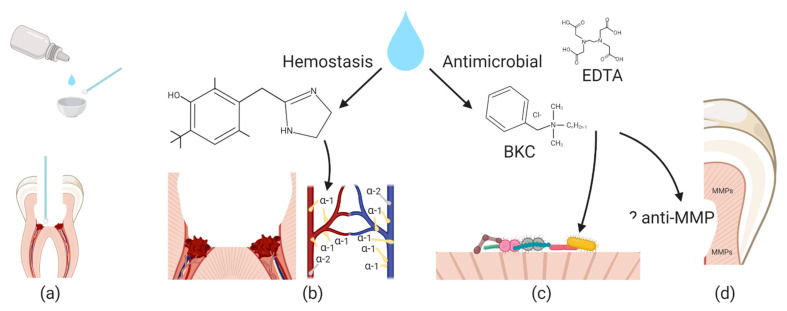
Conceptual Model of Imidazoline Activity. (**a**) Volume dosing of ImDs and use of microbrush to deliver solution to exposed pulp chambers. ImD solutions have possible multi-modal action (**b**) ImDs (oxymetazoline shown) are agonist to α-1 adrenoreceptors found in pulp tissue and innervation of these receptors can initiate vasoconstriction and hemostasis. (**c**) benzalkonium chloride (BKC) and EDTA work together to disrupt bacterial membranes and cationic properties can also aid in debris removal. Benzalkonium chloride is often a mixture of different chain length alkylbenzyldimethylammonium chlorides (*n* = 8, 10, 12, 14, 16, 18) (**d**) There is a hypothetical mechanism that BKC may inhibit dentine matrix metalloproteinase (MMPs) that can degrade a future dentin-adhesive layer. While hemostasis and antimicrobial action have extensive evidence, the MMP activity is based on limited studies and requires more investigation.

ImDs, such as oxymetazoline and xylometazoline, can be used in a dose controlled procedure to manage pulp tissue hemostasis, and commercial nasal (Afrin^®^, Bayer HealthCare LLC, Whippany, NJ, USA and Otrivine^®^, GlaxoSmithKline, Brentford, UK) solutions with ImDs also possess antimicrobial preservatives such as benzalkonium chloride (BKC) and EDTA that work together to potentially condition the cavity preparations surrounding the pulpal tissue (Figure 3). Additionally, BKC may also act as a matrix metalloproteinase (MMPs) inhibitor that may increase durability of long term dentin bonding agents used to restore the lost tooth structure [51].

Oxymetazoline and xylometazoline are agonists to α-1 adrenoreceptors, which are found in pulpal tissue and these receptors are primary mediators to sympathetic innervated vasoconstriction. Activation of α-1 adrenoreceptors by catecholamines will decrease flow of blood in pulpal tissue [52], and the use of oxymetazoline and xylometazoline is expected be more effective than epinephrine [17]. It is also expected that oxymetazoline and xylometazoline will be more effective than naphazoline and tetrahydrozoline based on the fact that the latter two ImDs are seldom used as hemostatic adjuncts in surgical procedures and have shorter duration of action. Oxymetazoline and xylometazoline are expected to have superior properties over other hemostatic agents. Oxymetazoline formulations have been used in animal and human gingival displacement studies and found to be more effective in temporary tissue shrinkage than epinephrine and aluminum chloride [6,8]. Sodium hypochlorite (NaOCL) is expected to be a slower hemostatic agent than ImDs, and NaOCL increases the pulpal inflammatory response [53,54]. For primary tooth pulpotomy management, the common hemostatic agent of 15% ferric sulfate (FeSO_4_) has an acidic pH of 1.6, and FeSO_4_ treated pulps are associated with internal resorption and post-operative pain [53,55,56]. Commercial nasal solutions of oxymetazoline and xylometazoline have a more biocompatible pH of 6.2–6.3 [55].

Benzalkonium chloride (BKC) is a quaternary ammonia compound with hydrophobic and cationic antimicrobial activity and often found in commercial formulations of 0.01–0.025%. BKC is biocompatible and often labeled as a single compound but can be a mixture of different chain length alkylbenzyldimethylammonium chlorides [57]. BKC has an extensive safety record and is well tolerated for both short and long term clinical use [58]. It has been added to dental adhesive products [51]. BKC has three potential roles for pulp exposure management. First, it is antimicrobial to Gram positive, and to a lesser degree Gram negative bacteria, at low concentrations [59]. BKC activity to penetrate and disrupt cell membranes is enhanced by the addition of EDTA in commercial nasal spray formulations [60,61]. Second, BKC is a cationic detergent that may condition the surrounding dentin tissue by removing bacteria and residue tissue. As a cation, it also has substantively by binding to demineralized tissue [51]. Third, BKC has been shown to be effective at inhibiting MMP activity (dentine matrix metalloproteinase (MMP) [51]. MMPs within untreated dentin may degrade the dentin-adhesive interface after resin composite placement. BKC which if applied to the pulpal tissue and the surrounding exposed dentin surface has the potential to improve dentin adhesive agents for composite restorations.

## 10. Discussion

This critical review is not a substitute for rigorous laboratory and clinical assessment of efficacy and safety for ImDs use in pulpal management. This review informs those considering laboratory and clinical investigations of ImDs in adults and children. ImD solutions are promising hemostatic and antimicrobial conditioning agents that could be more effective and biocompatible than existing agents in direct pulp capping in permanent and primary teeth, permanent teeth pulp management especially during apexiogenesis, and primary teeth pulpotomy procedures [62,63,64].

The review of the medical literature supports that oxymetazoline and xylometazoline are prospective 1st line ImDs for pulp management. This is based on their potency, duration of action, global availability, low cost, and extensive use for surgical hemostasis management in otolaryngology in both adults and children. Naphazoline and tetrahydrozoline are not commonly used for surgical hemostasis. It should be noted that while this critical review identifies oxymetazoline and xylometazoline as potential pulpal hemostatic agents, there is a need for discussing the important unanswered questions and the next steps for future research. While studies, including those on human gingival fibroblasts, support pulpal biocompatibility of IMDs, the dental pulp is a diverse and heterogeneous tissue composing of odontoblasts, fibroblasts, immune, mesenchymal, and dental pulp stem cells [7,41,42]. The effects of IMDs on each of these cell types warrants future investigation including investigating the osteogenic commitment capacity after ImDs exposure.

Future studies should directly compare oxymetazoline and xylometazoline with other ImDs and other current hemostatic agents. This also includes investigating the dose and volume need to establish hemostasis in specific situations of pulp capping, apexogenesis, and primary tooth pulpotomy. These investigations should consider the long term actions of IMDs and their effect on blood and lymphatic flow through the apical foramen. The question whether IMDs application can contribute to pulpal tissue necrosis risk has not been previously addressed and is a future area of study.

For researchers considering investigation, initial dose considerations of 0.05% oxymetazoline solutions (based on unpublished clinical outcomes), can be 0.015–0.05 mL of solution per tooth, with as little as 0.015 mL needed for single canal systems. Delivery can be achieved through the use of microbrush (~0.015 mL of solution) or defined volume soaking of small cotton balls (1 drop~0.05 mL) common in primary tooth pulpotomy procedures. As stated previously defined volume soaked pledgets on nasal mucosa had minimal systemic effects in children and it is postulated that there is a self-limiting bioavailability of oxymetazoline through local vasoconstriction [1,27]. This concept can be applied to pulpal tissue, where near immediate hemostasis of the small pulp exposure will limit systemic absorption of the ImD. Direct spraying of 0.05% oxymetazoline from the bottle into a pulpal access should be avoided since oral administration of ImDs, especially in children 5 and younger, can experience serious adverse events with ingestion of 1–2 mL [31,65].

Careful consideration needs to be taken for using ImDs in younger children 5 and younger, just as special precautions are done with local anesthetics. Future studies should examine this specific age group. It should be noted that for cases with numerous teeth needing pulp management during a single appointment visit, children are often managed under general anesthesia (GA). Anesthesia teams are experienced with IMDS such as oxymetazoline/xylometazoline and these are readily available in hospital settings. Dental care providers should communicate the use of ImDs to anesthesia providers who can monitor hemodynamic and pharmacokinetics effects of oxymetazoline/xylometazoline. It should be re-iterated that evidence from infants 1–12 months of age (mean~6 months of age) infants using single drop nasal application of 0.025% oxymetazoline in surgical settings produced no changes in hemodynamic parameters (BP, HR, respiratory rate) [25]. This emphasizes the importance of dosing similar to the use of local anesthetics in children. Another important consideration for children 5 and under, is that the several studies in otolaryngeal surgeries that suggest safety of ImDs in children are performing surgeries under GA where hemodynamic changes of oxymetazoline could be stabilized by effects of the general anesthetics themselves [1,2,24,25,26]. Further investigation into the pulpal use of ImDs in young children both inside and outside an operating room is needed. A potential advantage of using ImDs in large pediatric restorative cases performed under GA is that ImDs may shorten the overall time of gaining hemostasis over other existing compounds and thus shorten the time children are under the influence of GA.

The effects of the ImDs solution preservatives on oral bacteria and in dentine models should also be investigated. BKC has been kept at a concentration of 0.01–0.025% in these solutions to maintain tissue compatibility while also providing antimicrobial activity to residue bacteria. Studies examining sustained long term activity of BKC incorporated into biomaterials have shown effectiveness against oral microbes but these studies are not directly relatable to BKC found in commercial ImD solutions [66,67,68]. Future studies should examine the antimicrobial activity of commercial oxymetazoline and tetrahydrozoline solutions on in vitro oral multi-species bacteria models. The proposed anti-MMP activity to improve the dentine bonding effects should also be further investigated with in vitro dentine models.

Future experiments examining BKC and EDTA synergistic activity need to consider and document the specific brands of commercial ImD solutions used. The preservative ingredients may differ between brands and generic manufacturers. The surprising results that ciprofloxacin drops were equivalent to 0.05% oxymetazoline solutions in preventing infection otorrhea used specific brands (e.g., Afrin^®^) that may have specific preservative concentrations [43]. It is expected that most major brands have between 0.01–0.025% of BKC but other generic brands could potentially be lower or have less consistent concentrations between bottles. EDTA or sodium borate can be added to the solution at different concentrations as well to increase the efficacy of BKC. Nasal sprays differ from other types of cosmetic preservation since the spray bottles are continually challenged by microbes by the fact that insertion of the tip into the nose contaminates the bottle. Brand testing is needed when examining oral antimicrobial and anti-MMP activity.

The combination of BKC with EDTA found in commercial sprays may allow these solutions to cleanse the tooth cavity preparation, to have broad activity against to oral bacteria invading the deep dentinal tissue, and to have anti-MMP action in the surrounding dentine structure. Future research is needed to investigate the several possible modes of actions of oxymetazoline/xylometazoline commercial solutions in pulpal management and cross compare effectiveness and biocompatibility with existing topical dental hemostatic agents.

## 11. Conclusions

Alpha-adrenergic agonists, such as the ImDs oxymetazoline and xylometazoline, are highly effective hemostatic agents that may be more effective and biocompatible than existing pulp management compounds. With commercial nasal solutions also containing preservatives of benzalkonium chloride (BKC) and edetate disodium-EDTA, oxymetazoline and xylometazoline solutions may have potential to advance pulp management therapy through possible multi-modal action.

## Figures and Tables

**Table 1:** Commercially Available Solutions of Imidazoline Derivatives (ImDs) ^1^.

Compound	Primary Uses	USA Availability	Surgical Hemostasis	Duration (h)
0.05% Oxymetazoline HCL	Nasal Congestion	Yes	Yes	8
0.1% Xylometazoline HCL	Nasal Congestion	No	Yes	8
0.02% Naphazoline HCL	Conjunctivitis	Yes	No	4
0.1% Tetrahydrozoline HCL	Conjunctivitis	Yes	No	4

^1^ Micromedex (IBM). ImDs are available globally with some country specific regulations (USA shown).

**Table 2:** Medical Precautions and Drug Interactions with ImDs ^1^.

Medical Precautions		Drug Class Interactions
severe or unstable cardiovascular disease	uncontrolled hypertension or hypotension	fentanyl
		beta adrenergic antagonist
cerebral insufficiency	coronary disease	monoamine oxidase inhibitors (MAOIs)
Sjögren’s syndrome	narrow-angle glaucoma	
Raynaud’s phenomenon	thromboangiitis obliterans	tricyclic antidepressant (TCAs)
scleroderma	diabetes mellitus	
prostatic enlargement	thyroid disease	

^1^ Micromedex (IBM) of oxymetazoline, tetrahydrozoline HCL.

## Data Availability

No new data were created or analyzed in this study. Data sharing is not applicable to this article.
